# “You just need to leave the room when you breastfeed” Breastfeeding experiences among obese women in Sweden – A qualitative study

**DOI:** 10.1186/s12884-017-1656-2

**Published:** 2018-01-22

**Authors:** Ing-Marie Claesson, Lotta Larsson, Linda Steen, Siw Alehagen

**Affiliations:** 1 Department of Obstetrics and Gynecology, and Department of Clinical and Experimental Medicine, Linköping University, Linköping, Sweden; 2 University Hospital, Stavanger, Norway; 3 Department of Medical and Health Sciences, Linköping University, Linköping, Sweden; 4 Division of Obstetrics and Gynecology, Department of Clinical and Experimental Medicine, Faculty of Medicine and Health Sciences, Linköping University, SE - 581 83 Linköping, Sweden

**Keywords:** Breastfeeding, Experience, Obesity, Qualitative research

## Abstract

**Background:**

The benefits of breastfeeding for the infant as well for the mother are well-known. It is recognized that obese (Body Mass Index ≥30 kg/m^2^) women may have less antenatal intention to breastfeed, and shortened duration of breastfeeding compared with normal-weight women. This may result in adverse short- and long-term health for both mother and child, such as a shortened lactational amenorrhoea and decreased protection against breast cancer for the women, and an increased risk for infectious diseases and overweight/obesity among the children. Therefore, it is important to gain more knowledge and understanding of obese women’s experiences of breastfeeding in order to attain good health care. Hence, the aim of this study was to identify and describe obese women’s experiences of breastfeeding.

**Methods:**

This is an explorative study. Data was collected 2 – 18 months after childbirth through semi-structured face-to-face interviews with 11 obese women with breastfeeding experience. The interviews were recorded and transcribed verbatim. Thematic analysis was used.

**Results:**

Three themes emerged from the data analysis: Breastfeeding - a part of motherhood, the challenges of breastfeeding, and support for breastfeeding. The women described an antenatal hope for breastfeeding, the body’s ability to produce milk fascinated them, and the breast milk was seen as the best way to feed the child and also as promoting the attachment between mother and child. Breastfeeding was described as a challenge even though it is natural. The challenges concerned technical difficulties such as the woman finding a good body position and helping the child to achieve an optimum grip of the nipple. Another challenge was the exposure of the body connected to public breastfeeding. Support of breastfeeding was described as the importance of being confirmed as an individual behind the obesity, rather than an individual with obesity, and to obtain enough professional breastfeeding support.

**Conclusions:**

Breastfeeding was experienced as a natural part of being a mother. There were practical challenges for obese women concerning how to manage breastfeeding and how to handle the public exposure of the body. There was a need for realistic information about breastfeeding concerning both the child and the woman.

## Background

Worldwide obesity (Body Mass Index [BMI] ≥ 30 kg/m^2^) has more than doubled since the early 1980s and the prevalence among the female population, both across the world and in Sweden, is about 15% [1, 2]. Roughly 13% of pregnant women in the year 2015 in Sweden were obese at enrolment in antenatal care [3].

Pre-pregnancy obesity poses an increased risk for ante-, peri- and postnatal complications [4]. An excessive gestational weight gain may further worsen the situation [5]. Bever Babendure et al. [6] concluded in a novel review that obesity is a major risk factor for reduced initiation, duration and exclusively of breastfeeding. Mechanical factors such as additional tissue and larger breasts might be obstacles to breastfeeding, and furthermore, a protracted and complicated childbirth as well as postnatal edema are associated with delayed onset of lactogenesis II [6]. Previous studies have reported that, in comparison with normal-weight women, obese pregnant women seem to have less intention to breastfeed [7–9], whereas a recent study found no differences in antenatal intention for feeding [10]. Obese pregnant women with a recommended or excessive gestational weight gain have an increased risk of failing to initiate breastfeeding and of discontinuing exclusive breastfeeding, compared with normal-weight women with recommended weight gain [11]. However, a recently published study showed no differences in any or exclusive breastfeeding 3 months postpartum, according to gestational weight gain or pre-pregnancy BMI class [12]. Furthermore, fewer obese new mothers initiated breastfeeding and more obese women ceased within the first postnatal week, compared to normal-weight women [8, 13]. Overall, obese women have a shortened duration of breastfeeding in comparison with normal-weight women [8].

The relationship between obesity and psychosocial factors and its impact on breastfeeding initiation, exclusivity and duration has been shown in some studies. Obese breastfeeding women have a reduced confidence in their ability to reach their own breastfeeding goals. They also have few close friends and relatives who have breastfed and they experience lower social influence from others to breastfeed [14, 15]. Obesity has also been found to be associated with lower maternal self-efficacy and a higher rate of early breastfeeding cessation [16].They may encounter barriers to successful breastfeeding such as complications during the delivery, which might delay the establishment of skin-to-skin contact and by extension the initiation of breastfeeding, and may also cause sucking problems and difficulty positioning their infant [10, 15, 17, 18]. Other barriers may be lack of breastfeeding support, difficulty finding privacy in hospital or at home, and feeling uncomfortable when breastfeeding in public places [15, 18]. Additionally, body image concerns and body confidence may also affect the breastfeeding outcome. Great concern about the body image during pregnancy is associated with both intention to use and the actual use of formula, as well as shorter breastfeeding duration [19]. Overweight/obese women report more concerns with their body shape and, postnatally, are less confident about their bodies, compared with normal-weight women. Body concerns and lack of confidence and comfort are also associated with shorter lactation duration [20].

The benefits of breastfeeding for the child as well as for the mother are well-known. A recent meta-analysis [21] highlighted protection against infections and malocclusion, a lower risk of mortality, and a possible reduction in overweight and diabetes among children. Breastfeeding is associated with longer periods of amenorrhea, protection against breast cancer, and potentially also protection against ovarian cancer and type 2 diabetes [21]. Sweden as well as many other countries has adopted the recommendation of the World Health Organization of exclusive breastfeeding up to the age of 6 months, with continued breastfeeding along with appropriate complementary food during the first year of life, or as long as the parents and child want [22]. In the year 2015, a total of 95% of all children were breastfed at 1 week of age. Corresponding figures for four and 6 months of age were 74 and 63% respectively [23].

Low intention, failure initiation, and a short duration of breastfeeding among obese women may result in adverse short- and long-term health for both mother and child. Therefore, it is important to gain more knowledge and understanding of obese women’s experiences of breastfeeding in order to supply relevant health care. To our knowledge only a few qualitative studies have focused on lactating obese women, and to develop effective counselling and services for obese pregnant and breastfeeding women it is crucial to understand the phenomena from a woman’s point of view. Hence the aim of this study was to identify and describe their experiences of breastfeeding.

## Methods

### Study design, settings and participants

This was an explorative study, and data was collected through face-to-face interviews. The study was performed in the south-east of Sweden. Inclusion criteria for the study were: women with a self-reported pre-pregnancy weight and height calculated to BMI ≥ 30, normal pregnancy and childbirth, breastfeeding experience during the last 2-18 months, Swedish speaking and of European origin.

### Data collection

An interview guide was developed by the research group and consisted of eight open-ended questions (Table 1). The guide was tested by the two authors in two pilot interviews, which led to no change. The two interviews are therefore included in the data. All questions were covered in all interviews, but not necessarily in the same order, as the interviews followed the natural progression of a conversation.

**Table 1:** Interview guide

Can you tell me about:
1. Your expectations of breastfeeding during your pregnancy?
2. The first breastfeeding occasion?
3. Your experiences of breastfeeding during the maternity stay?
4. Your experiences of breastfeeding during the first period at home?
5. Your experiences of breastfeeding after a few months?
6. When you look back on your breastfeeding: What has been good/positive about breastfeeding your infant? What has been difficult?
7. Breastfeeding is associated with nudity and to some extent reveals a lot of skin. What are your experiences of this?
8. All participants in this study are women with obesity. Tell me about your thoughts about obesity associated with breastfeeding
Follow-up questions:
You say that …. Tell me more about …..
You say that …. Do you mean like this ….?
Can you give an example ….?

The participants were recruited using purposive sampling. Contact was made with the Weight Watchers Organization and instructors at three local offices were given oral and written information. The instructors then passed this information on to obese women who came to the local offices. A snowball inclusion [24] method was also used. Six women from the Weight Watchers and seven from the snowball inclusion were interested in participating in the study and they were given oral and written information. Two of the women did not fulfil the inclusion criteria, and a total of 11 women were interviewed.

All interviews were conducted by the two authors (LL, LS) between October 2014 and March 2015. The participants decided the date and place of the interview and most of them took place at the participants’ home but some of them in a public place (e.g. a cafeteria). The women were once again informed about the study and that they could withdraw their participation at any time, and also that the interview material would remain confidential and their identity would not be disclosed. Written informed consent was obtained from the participants. The interviews lasted for 30-41 min (median 35 min), and were digitally recorded and transcribed verbatim by LL and LS.

### Analysis

The data was analysed using inductive thematic analysis in accordance with Braun and Clarke [25] and it included six steps. (1) Two of the authors transcribed the interviews verbatim. The transcribed data was read several times by all the authors in order to gain familiarity. Initial ideas in the data were written down and discussed. (2) Initial codes were identified for features of the data that related to the study aim. The interviews were given equal attention and were coded for as many patterns as possible. (3) The codes were organized into initial themes by two of the authors, and then were revised jointly by all four authors. (4) The initial themes were reviewed in relation to the entire data. Coded extracts were moved to create coherent and consistent themes. Thematic maps were created to aid the generation of themes. (5) The themes were defined by identifying the core and writing down the content of each theme. All authors were active in refining the definitions and finally labelling the themes. (6) When writing the manuscript, citations were chosen to illustrate the findings.

### Ethics statement

The study was performed in accordance with the Declaration of Helsinki and Swedish legislation on non-invasive studies [26, 27].

## Results

Eleven obese women from south-east Sweden participated in the study. All characteristics were self-reported. The average age of the participants was 31 years (range from 24 to 40 years of age). Out of the 11 women, five were first-time mothers. BMI varied between 30.0 and 45.0 (median 31.2). Nine of the women had ceased breastfeeding at the time of the interview (the median of breastfeeding duration was 9 weeks) while two were still engaging in breastfeeding 3 months after childbirth. The characteristics of the participants are displayed in Table 2.

**Table 2:** Participant characteristics

Participant	Age	Body Mass Index	Parity	Highest Education Level Completed	Duration of Breastfeeding (weeks)
A	40	32.6	Multipara	Upper secondary school	8
B	33	30.1	Primipara	College or university	34
C	40	31.0	Multipara	College or university	56
D	25	31.2	Multipara	Upper secondary school	4
E	40	32.0	Primipara	College or university	4
F	30	31.1	Multipara	College or university	1
G	35	45.0	Multipara	Upper secondary school	> 12
H	26	31.0	Primipara	Upper secondary school	9
I	31	32.0	Primipara	Upper secondary school	38
J	30	30.0	Multipara	College or university	> 12
K	29	32.0	Multipara	Upper secondary school	23

**Fig. 1:**
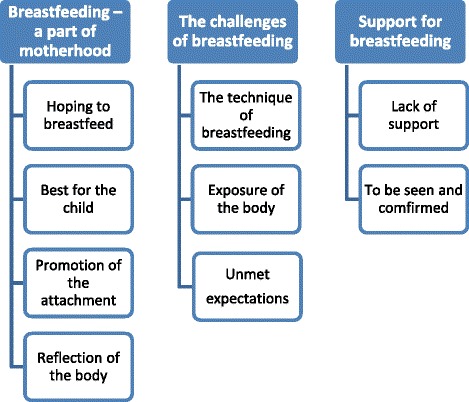
Main- and sub-themes

Obese women’s experiences of breastfeeding are described in three main themes and nine sub-themes (Fig. 1). The findings are exemplified with interview quotes termed A to K.

### Theme 1: Breastfeeding – A part of motherhood

The women stated that the motherhood was confirmed by the ability to breastfeed. Breastfeeding was stressed as something natural, and as obviously giving many advantages. It gave the possibility for closeness and confidence, and nutritionally, breast milk was seen as the best way to feed the child. Furthermore, it promoted the attachment between mother and child. The body’s own ability to produce milk and thus meet the needs of the child was experienced with fascination and joy. These factors were all reasons why the women chose breastfeeding.

#### Hoping to breastfeed

The breastfeeding expectations during the pregnancy were mostly described as positive. The women had an intention to breastfeed their children even if there was an underlying feeling that the breastfeeding might not work as expected.

“I thought after all that it was possible to breastfeed so I wanted to try, but I thought that if it didn’t work out it would be OK” (E)

Confidence in the ability to breastfeed was influenced by past experiences. Although breastfeeding had not worked with older children, there was still a hope that it would function properly this time.

“I had a hope that it would work. My plan was that I really wanted to breastfeed. That was my thought. I was quite prepared to do that. However, I was uncertain, given my past experiences, but my expectation and hope was that I could breastfeed” (A)

Factors such as theoretical knowledge about breastfeeding and participation in an antenatal education group contributed to a desire to breastfeed, but it also led to the realisation that not all women can breastfeed, and formula feeding was seen as an alternative.

#### Best for the child

The women considered that breastfeeding was a part of motherhood as breastmilk is nutritionally the best food for the child. They described positive properties of breast milk and also practical advantages of breastfeeding. The possibility to feed the child whenever there was a need for it helped them to continue being a feeding mother. The beneficial health effects of the breast milk also influenced the duration of the breastfeeding and was one of the factors that helped them to maintain breastfeeding to a greater extent than they had planned. The women also considered that there was a norm in society that a mother should breastfeed.

“They’re indoctrinated into that one should breastfeed one’s child …. breast milk is so amazingly good, it has all they need and they can take part of the mother’s immune system. Breast milk can be applied in the nose, eyes and ears as a kind of medicine and it will heal all kind of troubles. You feel some self-satisfaction being able to breastfeed your child because that is absolutely the best thing you can do for the child .... so in that way you become satisfied” (B)

#### Promotion of the attachment

The possibility to skin-to-skin-contact provided physical and emotional closeness. It provided the women from the beginning with an opportunity for natural nearness, facilitating the process of bonding. The women stated that breastfeeding gave them their priceless own time with the child which strengthened them in being a mother. Breastfeeding was highlighted as something more than just feeding and nutrition.

“In the beginning I found it perhaps a bit hard to bond. I had absolutely no postnatal depression or anything else, but I found bonding a bit difficult to understand in any way. When breastfeeding you are really close and I felt that I needed breastfeeding in order to bond properly. I would be special to him” (I)

“Closeness …. moments together … you always have the food with them ... peace and quiet … the contact we had together” (C)

#### Reflection of the body

The women described a positive experience when the child was suckling for the first time. The spontaneous and natural breast milk production created a feeling of satisfaction with the ability to be a nursing mother. Many women felt this satisfaction throughout the whole lactation period.

“Wow it works! One has heard from people that it will not start at once, haven’t you, but he needed only to suck twice and then the milk gushed from the breast and I thought that I was designed for breastfeeding” (H)

“He begins to suck directly! It is actually quite magical”. (I)

When the women reflected on the impact of body weight and breastfeeding, some of them said that a high BMI had a negative impact on breastfeeding, whereas others were not sure about it. There were those who compared themselves with others with well-functioning breastfeeding. They believed that lower body weight and smaller breasts could have a positive impact on breastfeeding.

“For them, it was just fine. The thought has crossed my mind that it could be due to the weight or size of the breast” (I)

### Theme 2: The challenges of breastfeeding

Even if there was an intention to breastfeed, the women stated that it was difficult during pregnancy to imagine the hard work required for well-working breastfeeding. The breastfeeding was often described as complicated and problematic and the women reported a number of difficulties they had to struggle with, mostly at the beginning. The joy and satisfaction with the ability to breastfeed was in contrast to the mental strain associated with public breastfeeding, due to showing their own body. When the women had been able to see the problems in perspective, some of them said the efforts to achieve functioning breastfeeding were worth it, although the opposite opinion also existed.

#### The technique of breastfeeding

The women described the difficulty of finding the right body position, giving the child the optimum grip on the nipple. For a number of women, to lie on one’s side was the best and the only breastfeeding position. This position made it hard to breastfeed in other places than in their own home and it created a feeling of isolation. This position also made the women miss the practical part of breastfeeding the child whenever the child needed it.

“I couldn’t sit up, I did not have a good technique and sometimes it was difficult to get it right, get the right grip and difficult to get started…” (F)

Women with large breasts talked about the child’s difficulties in getting a good grip on the nipple. The large breast leads to thoughts that the size may have a negative impact on the breastfeeding i.e. impaired milk production and wrong suckling grip. The wrong suckling grip leads to nipple lesions, which cause pain.

“I had very big boobs and that’s why I was thinking that they might not contain so much milk. It was impractical because he had difficulty getting the breast in his mouth so I thought it could have much to do with it” (I)

“They thought that I had such a heavy breast, which was why she had difficulty to grab. I would help to hold up the breast and I thought it worked better for a while, when I had done it. But then I thought surely it was too hard so I gave her formula and stopped breastfeeding” (E)

#### Exposure of the body

The women stated that at the time breastfeeding was carried out, both the woman’s body and the child were in focus. The breasts were more or less uncovered, which could arouse feelings of uncomfortableness in the breastfeeding woman, but it could also be a source of anxiety about negative reactions from people in the immediate environment. Public breastfeeding could therefore be a challenge for an obese woman. She faced the choice of customizing her clothing to minimize the exposure of the body or offering the child formula. Some women considered that they lost some of their confidence when they suddenly had to show their breast. This applied in public breastfeeding but also in health care situations when health care professionals (HPs) “poked and touched” the breast. They said that they had no option to say no. The women stated that obesity was often linked to low self-esteem and this influenced thoughts about breastfeeding and the practice of it.

“It has probably affected me so that I chose to bring formula because I knew that the situation would arise sooner or later. It was my safety valve to not have to breastfeed in public without being able to provide formula instead” (B)

“Maybe you are more ashamed to show your body while breastfeeding when you are overweight. You moved aside when you would breastfeed” (A)

#### Unmet expectations

When the breastfeeding did not reach the positive expectations which existed during pregnancy, negative emotion arose. If the child was unsatisfied during and after breastfeeding the women worried and experienced frustration, blame and negative stress. Failure to achieve the goal of functioning breastfeeding was described by some women as a defeat. Prematurely ceasing breastfeeding led to a bad conscience and afterwards a wish, despite tiredness and weakness, that they had fought more to achieve well-functioning breastfeeding.

“You did try to breastfeed and the result of it was that he cried and I cried….we struggled and struggled and struggled. Then followed an emotionally very tough period …. not being able to breastfeed” (D)

“With hindsight I would perhaps have tried a little more breastfeeding. But it’s not so easy. It’s easy to say in hindsight what one should have done” (F)

Breastfeeding was also described as a stressful and time-consuming process which influenced the whole family. The everyday pace was dragged down because breastfeeding was not going to speed up. Even though there was an awareness and acceptance that breastfeeding would take time to work optimally, some women felt as if they were continuously feeding their child.

“It was breastfeeding almost all the time. He ate and ate and ate…. so I felt like it was a marathon” (H)

### Theme 3: Support for breastfeeding

How well the breastfeeding works out can be influenced by the support available for the obese woman. HPs in the Maternity ward and Child Welfare Centre are critical supporters. To be confirmed as a breastfeeding mother in the beginning of the time with the child is crucial. Functional support can be described as holistic support including the needs of both the child and the woman.

#### Lack of support

The women talked about several challenges they had to face coming home with the new-born child. They struggled to help the child with gripping the nipple, gaining weight and helping the child to be satisfied with breastfeeding, but they also had to focus on themselves and issues such as like nipple lesions, tiredness, and fear of not managing to be the breastfeeding mother they were expected to be. The women often felt alone and vulnerable in the ongoing complex situation, of breastfeeding and being in transition to motherhood. They stated that it was important to meet an HP who could see both the child and the woman, and when necessary support the woman so she could succeed in breastfeeding according to her intention.

“It is very much preparations before a pregnancy and during a pregnancy afterwards you stand there without any handbook … you stand there very alone ” (A)

Some women stated that the support in the Child Welfare Centre mostly had focused on the child’s weight gaining, and the woman’s intentions or preferences for breastfeeding were in a way ignored. Thus, the women received recommendations about formula and often they lacked information about partial breastfeeding and support concerning how partial breastfeeding could proceed. This was one of the reasons why women ceased breastfeeding and some talked about having a bad conscience and disappointment about not being able to give the child the best nutrition. Many women said that these emotions were not noticed by the HP.

“In the Child Welfare Centre they thought that one should not spend any energy on breastfeeding … They told me to buy formula and I received no back up. I thought that should I do as they said. I couldn’t say or do anything else” (H)

Looking back at the time of decision-making regarding ceasing breastfeeding, some talked about it as a relief and others revealed disappointment. If breastfeeding was connected with negative stress including uncertainty about the child’s weight gain and an uncomfortable situation for the woman, ceasing breastfeeding was experienced as freeing. Others described frustration and stress of having to give up breastfeeding and connected it to lack of support and not having enough fighting spirit.

#### To be seen and confirmed

Many women stated that they received adequate and individual support. They experienced that HPs paid attention to them and their efforts to achieve functioning breastfeeding, confirming them and strengthening them in their new role as a breastfeeding woman. The support increased the women’s confidence in their ability to breastfeed, and being treated as an individual behind the obesity, rather than an individual with obesity strengthened self-confidence and also the trust in the healthcare system.

“When I was there with my first child, they often talked about my obesity and it was written 27 times that I was very overweight. This time they looked into my eyes and saw me as I was. Nobody focused on what I looked like. It is important that you receive the same information regardless of your weight.” (G)

## Discussion

The aim of the present study was to identify and describe obese women’s experiences of breastfeeding. The women described breastfeeding as a part of motherhood, as breast milk is the best for the child and breastfeeding promotes attachment. However, the breastfeeding experiences did not live up to the expectations they had during pregnancy. To breastfeed was a challenge both regarding the technique and the exposure of the body. The women described a need of support which confirmed them, and they wanted to be treated as an individual behind the obesity. They requested information about partial breastfeeding which might encourage them not to give up breastfeeding.

The results reveal that obese women’s experiences of breastfeeding in many ways are similar to those of normal-weight women, for example as regards breastfeeding as a natural part of motherhood, the challenges of breastfeeding and the need for support [28, 29]. According to the women in the present study, the challenges and the need of support were especially associated with the obesity and the body. Having big breasts was one reason that some mentioned as impaired or non-working breastfeeding. They compared their own breastfeeding situations with others who they thought had normal-sized breasts and a well-functioning breastfeeding. Some of the women expressed anxiety that big breasts could imply impaired milk production, and apprehensions about insufficient milk supply in obese breastfeeding women are also reported in other studies [17, 30, 31]. The women also described difficulties in finding a good breastfeeding position and helping the child to get a good grip of the breast nipple, and this was in agreement with other studies showing that large breasts and problems with the nipples may be associated with latching problems [4, 15].

The body was also in focus when talking about breastfeeding in public places. A majority of the women found this situation loathsome. The mentioned reasons the exposure of the breast as well as technical problems. This is in accordance with other studies [10, 18, 28, 30]. The need for privacy can ultimately lead to a shortened duration of breastfeeding and preference for formula, which is easier to handle in public. In addition, and from another perspective, lack of comfort or confidence with the body image is associated with reduced lactation duration [20] and may therefore contribute to choosing formula. The feelings of discomfort in public breastfeeding and a need for privacy can also be related to self-esteem, as high BMI is associated with lower self-esteem [32]. Previous studies have shown that overweight pregnant women feel stigmatised and exposed before they become pregnant [33–35]. The dissatisfaction with one’s body image seems to remain during pregnancy [36]. Higher body image concerns during pregnancy have been shown to be associated with formula use from birth, and with shorter breast feeding duration [37]. In a newly published study it was shown that women with obesity had poorer body image and were less likely to maintain breastfeeding compared with healthy weight women [38].

The public dilemma might result in social isolation, which was also mentioned by adolescent mothers who felt uncomfortable during public breastfeeding and thought it limited their possibilities to participate in social events [39]. Furthermore, the issue about public breastfeeding can be connected to attitudes in society about breastfeeding in public, and a study among New York residents showed that overall, 50% were not supportive of public breastfeeding [40].

The women described negative feelings when the breastfeeding did not reach the positive pre-pregnancy expectations. Similar findings have been reported in a meta-ethnographic synthesis of women’s experience of breastfeeding, and the terms ‘expectation’ and ‘reality’ were used [29]. Breastfeeding was not as easy as it looked and there was a sense of being disappointed with the reality [29]. The gap between expectations and experiences may result in premature cession of breastfeeding, which according to the participants in the present study, can be connected with a bad conscience for not having fought enough, but can also be a freeing decision. This finding is in line with findings in other studies showing that ceasing breastfeeding was often associated with feelings of failure and guilt about depriving the child of ‘the best’ but was also a crucial and necessary decision for the child’s health and well-being [29, 41]. The decision about giving up breastfeeding can be connected to the fact that during pregnancy the mothers had thought of this as natural and they were unprepared for the problems [41]. In a study by Brown [37], women stated that the antenatal care stressed mostly the health benefits, while the challenges of becoming and being a breastfeeding mother were hidden, and because of that they were not prepared for reality. In the present study, breastfeeding was described as a time-consuming and demanding process, and the same finding is reported in other studies where the breastfeeding process requires perseverance [15, 29]. The challenges of breastfeeding which the women in our study experienced illustrate that obese breastfeeding women should be offered additional and tailored breastfeeding support based on their individual needs. Some of the women in the present study stated that they had obtained professional advice and help, whereas others had experiences of loneliness in the situation. The HPs need knowledge and understanding about specific requirements as obese women seem to be less likely to seek postnatal breastfeeding support [18, 30].

Breastfeeding support includes many sources such as HPs, partners, parents and friends [28, 29] but the women in the present study focused on HPs and the need to be seen and confirmed. They had a need for individual support and to be seen as an individual behind the obesity. It is a challenge to give individual support and Swerts et al. [42] describe the midwife’s role in supporting breastfeeding using two different perspectives: the midwife as a source of technical support and as a skilled companion. According to that study, most of the women wanted support from a skilled companion but a majority of midwives provided support as a technical expert [42]. To support as a skilled companion might be a way to empower the obese breastfeeding women, as some women in our study said that support which confirmed them increased their confidence in their ability to breastfeed.

### Limitations and strengths

The limitation of this study is the small sample of women. The recommendation for sample size in a qualitative study according to Kvale and Brinkman [43] is 15 ± 10 depending on the aim of the study and the richness of information in the interviews. Our sample of mothers represents a variety of ages, which gives a variety of data, and according to Patton [24], variety strengthens a qualitative study.

A strength is that we used open-ended questions, and the first six were general and could also have been used for women with normal weight. Our intention with these questions was that the women would talk freely about their breastfeeding and not use the obesity as a label. The trustworthiness of the study was strengthened as the 15-point checklist of criteria for good quality of thematic analysis according to Braun and Clarke [25] was used.

Obesity has increased and in Western society the women’s body is in strong focus. Our results indicate that the body is a factor in the struggle with breastfeeding among obese women. Until now few studies have focused on experiences of breastfeeding related to the body. Therefore, it is important in future studies to endeavour to develop individual and effective counselling for pregnant and breastfeeding obese women.

## Conclusions and clinical implications

Breastfeeding was experienced as a natural part of motherhood. The knowledge about possibilities to offer the child the best nutrition was strengthened. However, there were challenges concerning practical aspects of how to manage breastfeeding and how to handle the public exposure of the body. There was a need for realistic information about breastfeeding and support for both the child and the woman. The women wanted to be seen as individuals behind the obesity.

These results might encourage HPs to reflect on how to objectively inform and prepare obese pregnant women for the challenges of the approaching breastfeeding, and how to provide skilled and individual breastfeeding support postnatally. There is a need to understand how obesity might influence the practical breastfeeding situation and women’s reactions to their bodies. This might be a way to decrease the gap between expectations and reality and to provide professional and ethical health-promotion support.

## Abbreviations

BMI: Body Mass Index
HP: Health care professional
